# Draft Genome Sequence of Antibiotic-Resistant Enterococcus faecalis Strain UMB0843, Isolated from the Female Urinary Tract

**DOI:** 10.1128/MRA.00407-20

**Published:** 2020-05-14

**Authors:** Natalia Purta, Taylor Miller-Ensminger, Adelina Voukadinova, Alan J. Wolfe, Catherine Putonti

**Affiliations:** a Neuroscience Program, Loyola University Chicago, Chicago, Illinois, USA; b Bioinformatics Program, Loyola University Chicago, Chicago, Illinois, USA; c Department of Microbiology and Immunology, Stritch School of Medicine, Loyola University Chicago, Maywood, Illinois, USA; d Department of Biology, Loyola University Chicago, Chicago, Illinois, USA; e Department of Computer Science, Loyola University Chicago, Chicago, Illinois, USA; Queens College

## Abstract

Here, we introduce the 2.8-Mbp draft genome of Enterococcus faecalis strain UMB0843, isolated from the female urinary tract. E. faecalis is a leading cause of nosocomial infections, and many strains are often resistant to multiple antibiotics. We focus our genome analysis on the multiple genes involved in antibiotic resistance in this strain.

## ANNOUNCEMENT

Enterococcus faecalis lives in the gastrointestinal tract of many organisms (1) and has recently emerged as a leading cause of nosocomial infections due to its increased resistance to many antibiotics (2, 3). E. faecalis is often a primary cause of surgical infections, infections within the bloodstream, and urinary tract infections (UTIs) (3, 4). Recently, we isolated E. faecalis strain UMB0843 from a urine sample obtained from a pregnant female. Here, we present the draft genome sequence of this isolate and the antibiotic resistance genes found within the genome.

E. faecalis UMB0843 was collected as part of a previous institutional review board (IRB)-approved study (5) and cultured using the expanded quantitative urine culture (EQUC) protocol (6). To determine the genus and species of each isolate, matrix-assisted laser desorption ionization–time of flight (MALDI-TOF) mass spectrometry was used, following a protocol detailed previously (6), prior to storage of the isolates at −80°C. From the freezer stocks, E. faecalis was streaked onto a Columbia nalidixic acid (CNA) agar plate and incubated at 35°C in 5% CO_2_ for 24 h. A single colony was selected, inoculated in brain heart infusion (BHI) broth (premixed BBL brain heart infusion; BD), and incubated under the same conditions described above. DNA was extracted using the Qiagen DNeasy blood and tissue kit and quantified using the fluorescence-based Qubit. The Gram-positive extraction protocol was followed with the following exception: 230 μl of lysis buffer was used (180 μl of 20 mM Tris-Cl, 2 mM sodium EDTA, and 1.2% Triton X-100 and 50 μl of lysozyme). The extracted DNA was sent to the Microbial Genome Sequencing Center (MiGS) at the University of Pittsburgh for sequencing. The DNA was first enzymatically fragmented using the Illumina tagmentation enzyme, and then indices were attached using PCR. Sequencing was performed using an Illumina NextSeq 550 flow cell, and 1,590,783 pairs of 150-bp reads were generated. The raw reads were trimmed using Sickle v1.33 (https://github.com/najoshi/sickle) and assembled using SPAdes v3.13.0 with the “only-assembler” option for k values of 55, 77, 99, and 127 (7). BBMap v38.47 (https://sourceforge.net/projects/bbmap/) was used to calculate the genome coverage. Genome sequences were annotated using PATRIC v3.6.3 (8), while the publicly available genome was annotated using the NCBI Prokaryotic Genome Annotation Pipeline (PGAP) v4.11 (9). Unless stated otherwise, default parameters were used for all software.

The E. faecalis UMB0843 draft genome is 2,805,168 bp long, assembled into 20 contigs with a genome coverage of 147× and an *N*_50_ score of 284,798 bp. The genome has a GC content of 38%, similar to that of other genomes of the species. PGAP identified 2,611 protein-coding regions. Both annotation tools found 51 tRNAs and 5 complete rRNA gene sequences (3 5S, 1 16S, and 1 23S). While resistance of this strain was not experimentally tested, PATRIC reported 39 genes associated with antibiotic resistance. Upon further investigation using ResFinder v3.2 (10), only antibiotic resistance to macrolides was detected [resistance gene, lsa(A)]. Our analysis suggests that this strain is susceptible to vancomycin, commonly used as the last line of defense. Vancomycin-resistant strains of E. faecalis emerged in the United States first in 1989 (11) and have become a significant concern for UTI treatment (12).

### Data availability.

This whole-genome shotgun project has been deposited in DDBJ/ENA/GenBank under the accession no. JAAUWL000000000. The version described in this paper is the first version, JAAUWL010000000. The raw sequencing reads have been deposited in the SRA under the accession no. SRR11441019.
